# Prevalence and predictors of binge eating disorder symptoms among a sample of university students in Bangladesh: A cross‐sectional survey

**DOI:** 10.1002/hsr2.1668

**Published:** 2023-10-31

**Authors:** Mohammad Tazrian Abid, Md. Hasan Al Banna, Shammy Akter, Keith Brazendale, Charles Spence, Mst. Umme Hafsa Begum, Rumana Rashid, Farzana Sultana Bari, M. A. Rifat, Mst. Sadia Sultana, Md. Khaleduzzaman, Sourav Chandra Debnath, Nushrat Mahjabin, Md. Shafiqul Islam Khan, Md. Nazmul Hassan

**Affiliations:** ^1^ Faculty of Nutrition and Food Science Patuakhali Science and Technology University Patuakhali Bangladesh; ^2^ Department of Food Microbiology Faculty of Nutrition and Food Science, Patuakhali Science and Technology University Patuakhali Bangladesh; ^3^ Nutrition Initiative Kushtia Bangladesh; ^4^ Department of Applied Nutrition and Food Technology Faculty of Biological Sciences, Islamic University Kushtia Bangladesh; ^5^ Department of Health Sciences University of Central Florida Orlando Florida USA; ^6^ Department of Experimental Psychology University of Oxford Oxford UK; ^7^ Department of Public Health Nutrition Primeasia University Dhaka Bangladesh; ^8^ Department of Global Public Health Karolinska Institutet Stockholm Sweden; ^9^ Department of Public Health and Informatics Jahangirnagar University Dhaka Bangladesh; ^10^ Department of Environmental Sanitation Faculty of Nutrition and Food Science, Patuakhali Science and Technology University Patuakhali Bangladesh

**Keywords:** Bangladesh, binge eating disorder, factors, prevalence, students

## Abstract

**Background and Aims:**

There is a dearth of information about binge eating disorder (BED) among Bangladeshi university students, who may be more susceptible to BED due to the rise in unhealthy lifestyles and food habits. Therefore, the purpose of this study was to assess the prevalence and associated factors of BED symptoms among Bangladeshi university students.

**Methods:**

Students (*N* = 525) from three public universities in Bangladesh participated in this cross‐sectional study between November 2022 and March 2023. Face‐to‐face interviews were conducted using a structured paper‐based questionnaire that included two validated survey tools; the binge eating disorder screener and the patient health questionnaire‐9. To identify the factors associated with BED symptoms, multiple logistic regression analysis was conducted, with sociodemographic and behavioral information (e.g., age, sex, smoking status, etc.) considered as covariates.

**Results:**

The prevalence of BED symptoms among participants (mean age 21.28 years, 50.3% male and 49.7% female) was 20.6%. Male students had a 2.28 times higher likelihood of having BED symptoms compared to female counterparts (adjusted odds ratio [AOR] = 2.28; 95% CI: 1.33−3.89). Older students (AOR = 3.56, 95% CI: 1.80−7.05), students who were overweight or obese (AOR = 3.32, 95% CI: 1.87−5.89), and students reporting higher depressive symptoms (AOR = 2.69, 95% CI: 1.66−4.35) were at greater risk for developing BED compared to their respective counterparts.

**Conclusions:**

This study provides new insights into the prevalence of BED symptoms and its contributing factors among Bangladeshi students. Approximately 1‐in‐5 university students reported having BED symptoms. University students who are older, overweight, or obese, and who report depressive symptoms may be at greatest risk. Future longitudinal studies are needed to determine the causal factors underlying BED. Findings from this study can assist policymakers and public health professionals in developing effective and targeted strategies to mitigate the risks associated with BED among Bangladeshi university students.

## 1 BACKGROUND

According to the DSM (*Diagnostic and Statistical Manual of Mental Disorders*)−5, criteria, binge eating disorder (BED) involves repeated episodes of excessive food consumption or indulging in large quantities for an extended duration.^1^ Individuals with BED often feel unable to control their consumption, eat rapidly resulting in excessive food intake in a relatively short period of time despite a lack of hunger which often results in discomfort. Individuals with BED also experience shame and guilt after a binge‐eating episode, which can lead to further binge‐eating episodes as a coping mechanism.^2^, ^3^ To meet DSM‐5 standards for BED diagnosis, episodes of binge eating must happen at least once a week and continue for a minimum of 3 months. Additionally, there must be associated feelings of distress related to the behavior.^4^

Studies indicate that BED is a common issue for people attempting to control their weight and enhance their general well‐being, with some studies reporting BED prevalence between 1.3% and 30.1%.^5^ BED is linked to a range of health physical and psychosocial health concerns^6^ such as weight gain, anxiety, depression, and low self‐esteem.^7^ Furthermore, individuals with BED have a higher probability of encountering other health complications including heart disease, diabetes, and high blood pressure. These physical and mental health problems can impact one's quality of life as it may contribute to social detachment, lack of drive, and trouble maintaining personal connections.^1^, ^3^ Other studies have also found that the severity of binge eating symptoms was positively correlated with the likelihood of suicide attempts and individuals with BED who had a history of suicide attempts were more likely to have comorbid psychiatric disorders, such as major depression and anxiety disorders.^8^ Thus, studies exploring the prevalence, and associated factors, of BED in populations who are at greater risk of developing a BED are of paramount importance to help inform public health professionals' development of interventions.

The prevalence of BED varies worldwide. For instance, the lifetime prevalence was found to be higher among females (2.8%) than males (1.0%) in France.^9^ In Portugal, the prevalence was found to be 9.6% among college students.^10^ In the United States of America, it was found at 2.8% among adults, and at a prevalence of 1.1% and 1.2% in Germany and Norway, respectively.^11^ In general, university or college students tend to have a higher prevalence of disordered eating than the general population, which can have detrimental effects on students' physical and mental health and well‐being, as well as their academic performance.^12^, ^13^

Currently, only a few published studies have been conducted among Bangladeshi students concerning the risk of eating disorders,^14^, ^15^ overweight and obesity and the risk of disordered eating,^16^ orthorexia nervosa with other factors,^17^ and association between eating disorder and modern culture.^18^ However, no studies have specifically explored the symptomology of BED among the university student populations in Bangladesh. A comprehensive investigation into the prevalence and factors associated with BED among university students in Bangladesh is crucial for several reasons. University students represent a distinct and vulnerable demographic group characterized by various academic, social, and lifestyle challenges. These challenges often converge, creating a unique environment that can significantly impact students' eating behaviors, physical, and mental well‐being. In addition, the rise in fast‐food establishments, especially on university campuses, along with their affordable prices has made it incredibly convenient for students to indulge in quick and varied meal options. This shift can be attributed to changes in lifestyle and cultural preferences among students who prioritize convenience and speed over traditional home‐cooked meals. BED impact students' academic achievement, personal relationships, and overall life satisfaction.^19^ Given the increased vulnerability to developing BED among university students and the lack of relevant published literature in the country, the present study is designed to bridge the research gap among this vulnerable group. Hence, the purpose of the present study was to investigate the prevalence and factors related to BED symptoms among students attending universities in Bangladesh.

## 2 METHODS

### 2.1 Study design, participants, and procedure

This cross‐sectional study was conducted between November 2022 and March 2023. The sample was recruited from public universities (*n* = 3) in Bangladesh. A minimum sample of 168 was calculated using a single population proportion test by assuming a 12.5% prevalence of BED symptoms among Bangladeshi university students based on the findings of pilot testing (*n* = 32), a 95% level of precision, and a 5% margin of error.^20^ The study recruited more participants than the estimated sample size to compensate for participant nonresponses and to increase the external validity and generalizability of the study findings.^21^ With incomplete or ineligible data excluded, 525 respondents' responses were included in the final analyses, out of 540 students from three public universities in Bangladesh.

The participants were recruited using a simple random selection method. Five field investigators visited the three campus sites to recruit study participants by canvassing. The field investigators approached students on the campuses at random, gave them an explanation of the study's purpose, and assessed study eligibility criteria from interested students. For study eligibility, the participants had to be a full‐time enrolled student and 18 years or older. Participants were excluded if they had a clinically diagnosed eating disorder or serious health condition, such as diabetes and hypertension.

Survey data were collected through face‐to‐face interviews using a structured paper‐based questionnaire (Supporting Information File). The interviewee read each item on the questionnaire with all possible response options to the participant and filled it out in person. Each interview took approximately 12−15 min to complete. Written informed consent was obtained from the study participants before starting data collection. Participation in this study was self‐nominated, and no incentives were offered to the survey participants.

### 2.2 Variables

There were 10 variables in this study, including one outcome variable and nine independent variables.

#### 2.2.1 Outcome variable

The symptoms of BED was assessed using the seven‐item binge eating disorder screener (BEDS‐7).^22^ The BEDS‐7 was validated according to DSM‐5 diagnostic criteria with a sensitivity of 100% and specificity of 38.7%.^22^, ^23^ Initially, the following screening question (yes vs. no) was asked to assess whether the participants had engaged in excessive eating over the past 3 months, “*During the last 3 months, did you have any episodes of excessive overeating (i.e., eating significantly more than what most people would eat in a similar period of time)*?” If the participant responded positively (i.e., “yes”) to the screening item, they were asked whether they felt distressed from their episodes of excessive overeating (item number 2: yes vs. no). The subsequent five questions (item numbers 3−7) used a Likert‐like rating scale (never/rarely, sometimes, often, or always) to measure other relevant factors. The presence of BED symptoms were indicated by a positive response (i.e., “yes”) to each of the first two questions, an affirmative answer of “always,” “often,” or “sometimes” to items three through six, and a response of “never or rarely” or “sometimes” to item seven.^22^

#### 2.2.2 Independent variables

The patient health questionnaire‐9 (PHQ‐9), which consists of nine distinct questions, was used to measure depressive symptoms.^24^ Each response was coded on the PHQ‐9 scale from “not at all” (0 points) to “nearly every day” (3 points). The overall score [0−27] was divided into two categories. Scores of 10 or more indicated depressive symptoms, and scores of less than 10 indicated no signs of depressive symptoms.^25^, ^26^ This scale is a valid and reliable measure of depressive sympotms, and has been used among Bangladeshi university students in previous studies.^27^

Moreover, participants' sociodemographic and behavioral information such as sex (male vs. female), age, study major (engineering, health science, biological science, business studies, or others), marital status (unmarried vs. married), monthly family income, smoking status (yes vs. no), self‐reported BMI status (underweight, normal weight vs. overweight/obese), and physical activity level (physically inactive, moderate physical activity, regular physical activity, or regular extensive activity) were captured as covariates. During the survey, participants' ages and monthly family income were recorded as continuous measures, and these variables were then classified as “18−20 versus 21−23 versus ≥24 years” for age and “≤30,000 Bangladeshi taka BDT versus >30,000 BDT” for family income.

### 2.3 Data analysis

Enumerative statistics such as responses and percentages were computed to summarize study variables. A *χ* ^2^ test was performed to observe the distribution of BED across the explanatory variables. A multiple logistic regression model was fitted to identify the factors associated with BED. All explanatory variables (*N* = 9) were included in the adjusted regression model to assess the estimated adjusted effects of the factors associated with BED symptoms. The fitness of the adjusted regression model was checked by the Hosmer and Lemeshow goodness of fit test. Odds ratios with 95% confidence intervals (CI) were calculated for both the adjusted and unadjusted regression models. An odds ratio plot was created for the graphical presentation of the findings of the adjusted regression analysis. The cut‐off value for statistical significance was set at <0.05 (two‐tailed). All analyses were performed using the STATA (BE version 16.0; StataCorp.).

## 3 RESULTS

### 3.1 Sample characteristics

The study sample comprised of 525 students (male: 50.3%, female: 49.7%) with a mean age of 21.28 years (SD: 1.61, age range: 18−27 years). Other background information and distribution of BED symptoms based on participants' characteristics are shown in Table 1.

**Table 1:** Distribution of binge eating disorder symptoms (yes vs. no) based on participants' characteristics (*n* = 525).

Variable(s)	Total	Binge eating disorder		*χ* ^2^ statistic	
	Frequency (%)	Yes	No	*χ* ^2^ value (*df*)	*p* Value
Sex				6.37 (1)	**0.012**
Male	264 (50.3)	66 (25.0)	198 (75.0)		
Female	261 (49.7)	42 (16.1)	219 (83.9)		
Age (in years)				17.65 (2)	**<0.001**
18−20	165 (31.4)	29 (17.6)	136 (82.4)		
21−23	309 (58.9)	57 (18.4)	252 (81.6)		
≥24	51 (9.7)	22 (43.1)	29 (56.9)		
Study major				1.39 (4)	0.846
Engineering	80 (15.2)	15 (18.8)	65 (81.3)		
Health science	81 (15.4)	19 (23.5)	62 (76.5)		
Biological science	163 (31.0)	36 (22.1)	127 (77.9)		
Business studies	76 (14.4)	13 (17.1)	63 (82.9)		
Others	125 (23.8)	25 (20.0)	100 (80.0)		
Marital status				0.05 (1)	0.828
Unmarried	498 (94.9)	102 (20.5)	396 (79.5)		
Married	27 (5.1)	06 (22.2)	21 (77.8)		
Family income (BDT/month)				0.08 (1)	0.927
≤30,000	318 (60.6)	65 (20.4)	253 (79.6)		
>30,000	207 (39.4)	43 (20.8)	164 (79.2)		
Smoking status				5.73 (1)	**0.017**
Yes	47 (9.0)	16 (34.0)	31 (66.0)		
No	478 (91.0)	92 (19.2)	386 (80.8)		
Self‐reported BMI status				20.13 (2)	**<0.001**
Underweight	80 (15.2)	21 (26.3)	59 (73.8)		
Normal weight	352 (67.0)	54 (15.3)	298 (84.7)		
Overweight/obese	93 (17.7)	33 (35.5)	60 (64.5)		
Physical activity level				1.77 (3)	0.621
Physically inactive	230 (43.8)	52 (22.6)	178 (77.4)		
Moderate physical activity	204 (38.9)	41 (20.1)	163 (79.9)		
Regular physical activity	49 (9.3)	09 (18.4)	40 (81.6)		
Regular extensive activity	42 (8.0)	06 (14.3)	36 (85.7)		
Depressive symptoms				21.45 (1)	**<0.001**
Yes	209 (39.8)	64 (30.6)	145 (69.4)		
No	316 (60.2)	44 (13.9)	272 (86.1)		

*Note*: Bold Values are statistically significant.

Abbreviations: BDT, Bangladeshi taka (currency); BMI, body mass index; df, degree of freedom.

### 3.2 Prevalence and predictors of BED

According to the BEDS‐7, the prevalence of BED symptoms among study participants was 20.6% (95% CI: 17.1−24.0). *χ* ^2^ test found that participants' gender (*p* = 0.012), age (*p* < 0.001), smoking status (*p* = 0.017), BMI status (*p* < 0.001), and depressive symptoms (*p* < 0.001) were significantly associated with the distribution of BED symptoms (yes vs. no) (see Table 1).

The unadjusted regression analysis found that the following factors were associated with BED symptoms: (i) being male (crude odds ratio [COR] = 1.7; 95% CI: 1.13−2.68), (ii) being aged 24 years or older (COR = 3.56; 95% CI: 1.80−7.05), (iii) being a smoker (COR = 2.17; 95% CI: 1.14−4.13), (iv) being underweight (COR = 1.96; 95% CI: 1.10−3.50) and overweight/obese (COR = 3.04; 95% CI: 1.81−5.08), and (v) having depressive symptoms (COR = 2.73; 95% CI: 1.77−4.21) (see Table 2).

**Table 2:** Unadjusted binary regression analysis demonstrating the factors associated with the symptoms of binge eating disorder among study participants (*n* = 525).

Variable(s)		Unadjusted regression model		
		95% confidence interval		
	Crude odds ratio	Lower Limit	Upper Limit	*p* Value
Sex				
Male	1.7	1.13	2.68	0.012*
Female	Reference			
Age (in years)				
18−20	Reference			
21−23	1.06	0.65	1.74	0.815
≥24	3.56	1.80	7.05	<0.001*
Study major				
Engineering	0.92	0.45	1.88	0.826
Health science	1.23	0.62	2.41	0.555
Biological science	1.33	0.64	2.01	0.668
Business studies	0.83	0.39	1.73	0.612
Others	Reference			
Marital status				
Unmarried	0.90	0.35	2.91	0.828
Married	Reference			
Family income (BDT/month)				
≤30,000	Reference			
>30,000	1.02	0.66	1.57	0.927
Smoking status				
Yes	2.17	1.14	4.13	0.019*
No	Reference			
Self‐reported BMI status				
Underweight	1.96	1.10	3.50	0.022*
Normal weight	Reference			
Overweight/obese	3.04	1.81	5.08	<0.001*
Physical activity level				
Physically inactive	Reference			
Moderate physical activity	0.86	0.54	1.37	0.525
Regular physical activity	0.77	0.35	1.69	0.515
Regular extensive activity	0.57	0.23	1.43	0.231
Depressive symptoms				
Yes	2.73	1.77	4.21	<0.001*
No	Reference			

*Note*: Bolded and asterisk values indicate statistically significant (i.e., *p* < 0.05).

Abbreviations: BDT, Bangladeshi taka (currency); BMI, body mass index.

**Figure 1:**
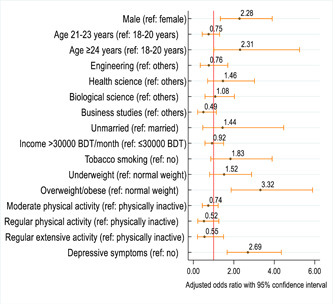
Odds ratio plot (with corresponding 95% confidence interval [CI]). This plot shows the factors associated with binge eating disorder symptoms among study participants (adjusted regression model). The circle marker and horizontal orange range of each row represent a specific variable's odds ratio and 95% CI, respectively. The vertical red line of the plot region represents the reference line for odds ratio (odds ratio = 1), and the variables were considered statistically significant when the associated 95% CI did not cross the reference line.

The adjusted estimated effects of the factors associated with the BED symptoms among study participants are depicted in Figure 1. The adjusted regression analysis showed that the likelihood of developing BED symptoms was two times higher among male students than their female counterparts (adjusted odds ratio [AOR] = 2.28; 95% CI: 1.33−3.89). Students ≥24 years were more likely to develop BED symptoms compared to their counterparts (AOR = 3.56; 95% CI: 1.80−7.05). Those students who classified themselves as overweight/obese had a three times higher likelihood of developing BED symptoms as compared to their counterparts (AOR = 3.32; 95% CI: 1.87−5.89). Those students who had depressive symptoms were more likely to develop BED symptoms as compared to their counterparts (AOR = 2.69; 95% CI: 1.66−4.35) (see Figure 1 and Table 3).

**Table 3:** Adjusted binary logistic regression analysis demonstrating the factors associated with binge eating disorder symptoms among study participants (*n* = 525).

		Adjusted regression model		
		95% confidence interval		
Variable(s)	Odds ratio	Lower limit	Upper Limit	*p* Value
Sex				
Male	2.28	1.33	3.89	0.003*
Female	Reference			
Age (in years)				
18−20	Reference			
21−23	0.75	0.44	1.29	0.304
≥24	2.31	1.01	5.25	0.047*
Study major				
Engineering	0.76	0.34	1.69	0.507
Health science	1.46	0.71	3.02	0.307
Biological science	1.08	0.57	2.02	0.814
Business studies	0.49	0.22	1.13	0.095
Others	Reference			
Marital status				
Unmarried	1.44	0.47	4.46	0.526
Married	Reference			
Family income (BDT/month)				
≤30,000	Reference			
>30,000	0.92	0.57	1.49	0.745
Smoking status				
Yes	1.83	0.86	3.89	0.115
No	Reference			
Self‐reported BMI status				
Underweight	1.52	0.79	2.88	0.202
Normal weight	Reference			
Overweight/obese	3.32	1.87	5.89	<0.001*
Physical activity level				
Physically inactive	Reference			
Moderate physical activity	0.74	0.44	1.22	0.238
Regular physical activity	0.52	0.22	1.26	0.148
Regular extensive activity	0.55	0.20	1.49	0.240
Depressive symptoms				
Yes	2.69	1.66	4.35	<0.001*
No	Reference			
Model summary	*χ* ^2^ value	(*df*)		*p* Value
Hosmer and Lemeshow test	9.506	8		0.301

*Note*: Bolded and asterisk values indicate statistically significant (i.e., *p* < 0.05).

## 4 DISCUSSION

The study presented here was designed to investigate the prevalence and factors associated with BED symptoms among Bangladeshi university students. The findings revealed that approximately two in every 10 Bangladeshi university students (20.1%) in our study sample exhibited BED symptoms. Furthermore, Bangladeshi students who were male, older (age ≥24 years), overweight or obese, and depressed were at greater risk of having BED symptoms. The findings derived from our study highlight the multifaceted nature of BED among Bangladeshi university students, emphasizing the importance of implementing holistic interventions that consider factors such as age, weight status, and depression to effectively address its prevalence and hence to combat BED.

The prevalence of BED symptoms can vary across different populations and studies. The present study's prevalence is much higher than a community‐based study conducted by Hudson et al. in 2012,^28^ which included a large sample of community adults (age range of 18−70 years) in the United States (2.9% in women and 3.0% men). However, in another study by Lipson et al., a BED prevalence rate of 40.2% was reported among undergraduate and graduate students in the United States.^29^ Grilo et al. also investigated the prevalence of BED among college students in United States and reported a prevalence rate of approximately 20%, which aligns with our findings.^30^ It is essential to acknowledge that the prevalence of BED varies across different populations and studies, and the prevalence reported in the present study falls within this range, indicating the relevance of our findings for similar student populations.^31^ However, it is important to note that our study focused on a particular group of individuals enrolled in universities, whereas Hudson et al.^28^ focused on a community‐based sample of adults that ranged in age from 18 to 70 years. Variations in sample characteristics, such as age range, gender distribution, and inclusion criteria, can contribute to the differences in prevalence rates. In addition, variations in prevalence rates across studies can be influenced by sample characteristics, assessment methods, diagnostic criteria used, and also potential geographical differences.^3^ Nonetheless, these findings add to the growing body of literature on the prevalence of BED among adults, specifically those who attend university (i.e., young adults), and may help prioritize specific populations (i.e., young adults) for intervention.

Other notable findings emerged such as that males were identified as having an increased risk of BED symptoms compared to females. This result is consistent with several previous studies that have reported a higher prevalence of BED amongst males in the same age group. Previous studies^31^, ^32^ found that males were more likely to exhibit binge eating behaviors than females. In contrast to these findings, one study conducted in France stated that the lifetime prevalence of BED was higher among females than amongst males.^9^ The way in which society views body image, weight stigma, and cultural norms regarding eating habits can greatly impact the development of BED in males versus females. It is apparent that extreme dieting and purging have risen more rapidly amongst men than women due to societal expectations. This demonstrates a notable discrepancy between how BED manifests itself in the two sexes.^33^ Previous research has suggested that males may experience unique challenges and pressures with regard to their body image, which can contribute to the development of BED and other disordered eating behaviors.^34^, ^35^ Additionally, the underdiagnosis or misdiagnosis of BED in males due to gender‐specific biases in clinical settings could also contribute to the observed higher prevalence.^32^

The findings stated that participants aged ≥24 years had a significantly higher risk of BED symptoms compared to younger counterparts. Previous research has found that BED prevalence tends to increase with age.^36^ This trend could be attributed to various factors, such as changes in life circumstances, increased stressors, and body image concerns that may manifest or intensify with age. The transition from undergraduate to graduate life often involves significant challenges such as the final push to graduate and postgraduation planning. Previous studies have reported the prevalence of depression and stress is high among graduate students.^37^ These stressors can be an influencing factor of a person's emotional well‐being and eating habits, which could contribute to the heightened risk of BED seen in this demographic. Further, eating disorders, such as BED, may be under diagnosed among older adults due to lack of awareness and education among primary care physicians treating this age group.^38^ Older adults with eating disorders may also find increased barriers and access to treatment due to limited income and inadequate health insurance coverage.^38^

A significant relationship between self‐reported BMI status and BED symptoms was found in the present study. Bangladeshi university students classified as overweight or obese, and underweight individuals, had increased odds of BED symptoms compared to those with normal weight. These findings are consistent with previous research that has identified BMI as a potential risk factor for BED. For instance, a study by Grilo et al. investigated that higher BMI was associated with increased overvaluation of shape and weight, suggesting a link between higher BMI and the presence of BED symptoms.^30^ It is important to note that the direction of causality between BMI and BED is complex and can be bidirectional. While higher BMI may contribute to the development or exacerbation of BED, the presence of BED symptoms can also influence changes in weight and BMI. These may be because the presence of excess weight and body dissatisfaction may contribute to the development and maintenance of disordered eating patterns, including binge eating behaviors.^39^ Furthermore, our findings emphasize the importance of acknowledging that individuals who indicate that they are underweight also face a potential risk for BED symptoms. A study by Walsh and Devlin highlighted that in the past, eating disorders such as BED had been commonly linked to individuals who are underweight or have a normal weight.^40^ Further longitudinal studies can be conducted to investigate the complex and bidirectional relationship between BMI and BED symptoms to get a more comprehensive grasp of its causal factors and potential consequences.

Study participants with depressive symptoms had a higher odds of BED symptoms compared to those without depressive symptoms, which aligns with previous research.^41^, ^42^ This suggests that depressive symptoms independently contribute to the risk of BED. The presence of depressive symptoms may be indicative of underlying psychological distress, emotional dysregulation, or coping difficulties that can increase vulnerability to disordered eating behaviors, including binge eating and the presence of BED symptoms can also contribute to depressive symptoms through feelings of guilt, shame, and low self‐esteem associated with the disorder.^43^ The interaction between depressive symptoms and BED symptoms highlighted the bidirectional relationship, whereby each can serve as both a cause and a consequence of the other.

There are several key takeaways from the findings of the present study. To effectively reduce the risk of developing BED amongst Bangladeshi university students—and the subgroups who may be at higher risk of BED (e.g., students who are male, older, have a non‐normal BMI, exhibit depressive symptoms)—strategies and initiatives are needed across university programs and campuses that promote healthy eating habits and prioritize emotional well‐being. Moreover, given the relationship between depressive symptoms and BED symptoms, it becomes imperative to offer comprehensive mental health services tailored specifically for university students. Efforts should focus on destigmatizing seeking help for mental health concerns as well as improving access to counseling and psychological support services at campuses. Additionally, considering the correlation between BMI status and BED symptoms, adopting a holistic approach toward body image perception and weight management is an important consideration. Campus healthcare centers can play a key role by promoting an overall sense of well‐being rather than solely emphasizing weight loss or appearance.

### 4.1 Study strengths and limitations

Strengths of this study include the large sample size and recruitment from three different university campuses. The use of valid and reliable measures of BED symptoms and associated factors is an additional strength. Furthermore, being one of the first studies in Bangladesh, this study provides preliminary data on the symptomology of BED to strengthen future research and policy initiatives. Nonetheless, there are limitations that must be acknowledged. The present study incorporated a cross‐sectional approach, thus a causal relationship between BED and associated factors cannot be determined. Since the study sample was drawn from three public universities in Bangladesh, the findings cannot be generalized to other contexts such as private universities or other age groups. Lastly, although the surveys incorporated in this study have adequate reliability and validity, BEDS‐7 assesses the symptomology of BED rather than making a clinical diagnosis, and the self‐reported nature of these assessments does not rule out response biases. Furthermore, responses could be impacted by information bias because of cultural aspects. For example, in Bangladesh, it is very common that in social ceremonies, peoples/guests are requested to eat more and meeting those requests are considered as social norms. In these instances, the overall prevalence of BED symptoms could be overestimated; however, the likelihood of such cases in this study is low, especially for participants who are resident students.

## 5 CONCLUSIONS

Our study findings revealed that approximately 1‐in‐5 Bangladeshi university students have BED symptoms and certain subgroups are at an increased risk of BED. These included male students, those who were 24 years old or older, those classified as underweight, overweight, or obese, and those individuals exhibiting depressive symptoms. The present findings highlight the need for awareness campaigns and educational programs targeting university students, with a particular focus on vulnerable groups. Such initiatives could help to raise awareness about the risks associated with BED and improve early detection and intervention. It is imperative to ensure that mental health services are readily available for students, placing special importance on addressing issues related to body image, depressive symptoms, and stress management. Additionally, it is crucial to conduct further studies to examine the cultural and societal aspects that may contribute to the prevalence of BED symptoms amongst university students from Bangladesh. Longitudinal studies can help establish causal relationships between the identified factors and BED, providing insights into potential preventive measures. Understanding the unique challenges faced by vulnerable groups can help to inform the design of tailored intervention strategies to reduce the burden of BED in these populations. Further studies are warranted to investigate the underlying mechanisms behind the identified relationships and to assess the efficacy of intervention strategies designed specifically for university students at risk of developing BED.

## AUTHOR CONTRIBUTIONS

**Mohammad Tazrian Abid**: Conceptualization; data curation; investigation; project administration; writing—original draft. **Md. Hasan Al Banna**: Conceptualization; formal analysis; investigation; methodology; project administration; software; supervision; writing—original draft; writing—review and editing. **Shammy Akter**: Resources; validation; visualization; writing—review and editing. **Keith Brazendale**: Validation; visualization; writing—review and editing. **Charles Spence**: Validation; visualization; writing—review and editing. **Mst. Umme Hafsa Begum**: Validation; visualization; writing—review and editing. **Rumana Rashid**: Validation; visualization; writing—review and editing. **Farzana Sultana Bari**: Validation; visualization; writing—review and editing. **M. A. Rifat**: Visualization; writing—original draft; writing—review and editing. **Mst. Sadia Sultana**: Validation; visualization; writing—review and editing. **Md Khaleduzzaman**: Data curation; investigation; writing—original draft. **Sourav Chandra Debnath**: Data curation; investigation; writing—original draft. **Nushrat Mahjabin**: Data curation; investigation; writing—original draft. **Md. Shafiqul Islam Khan**: Supervision; validation; visualization; writing—review and editing. **Md. Nazmul Hassan**: Supervision; validation; visualization; writing—review and editing.

## CONFLICT OF INTEREST STATEMENT

The authors declare no conflict of interest.

## ETHICS STATEMENT

This study was conducted following the Declaration of Helsinki, and the study protocol was reviewed and approved by the Research Ethical Committee of the Department of Environmental Sanitation, Patuakhali Science and Technology University, Bangladesh (ENS: 15/10/2022:09).

## TRANSPARENCY STATEMENT

The lead author Md. Hasan Al Banna affirms that this manuscript is an honest, accurate, and transparent account of the study being reported; that no important aspects of the study have been omitted; and that any discrepancies from the study as planned (and, if relevant, registered) have been explained.

## Supporting information

Supporting information.

## Data Availability

The data will be shared upon request to the corresponding author.
