# Supplementary material for: Prevalence and predictors of binge eating disorder symptoms among a sample of university students in Bangladesh: A cross‐sectional survey
Source: Health Sci Rep. 2023 Oct 31;6(11):e1668. doi: 10.1002/hsr2.1668 (PMC10618435; doi:10.1002/hsr2.1668)
Supplement: Supplementary file 1 — Supporting information. [file HSR2-6-e1668-s001.docx]

**Annexure 1: Questionnaire**


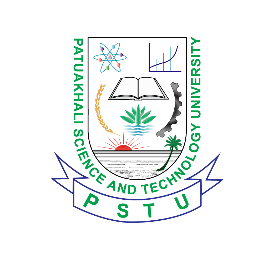
Serial number: ……….

**Survey Questionnaire**

**Title: Binge eating disorder among university students in Bangladesh**

**Part 1: Socio-demographic and related characteristics of the study participants**

| 1. What is your gender? | 1. Male (b) Female |
| --- | --- |
| 1. What is your age (years)? | ………………. |
| 1. Educational Background | (a) Engineering (b) Health science (c) Life/Biological sciences  (d) Business studies (e) others |
| 1. Marital status | (a) Unmarried (b) Married |
| 1. Household income | …………………. |
| 1. Do you smoke? | (a) Yes (b) No |
| 1. Self-perceived BMI status | (a) Underweight (b) Normal weight/healthy (c) Overweight or Obese |
| 1. Physical Activity Level | (a) Physically inactive/ almost completely inactive - watching TV, reading books, using computers  (b) Moderate physical activities are cycling or walking to work, or gardening.  (c) Regular physical activity (at least 2-3 hours a week) - swimming, running, having a laborious garden....  (d) Regular difficult physical activity for sports/competition (a few times a week) - swimming, running, |

**Part 2: The status of depression (PHQ-9)**

| **Statement** | Not at all (0) | Several days (1) | More than half the days (2) | Nearly every day (3) |
| --- | --- | --- | --- | --- |
| 1. Little interest or pleasure in doing things |  |  |  |  |
| 2. Feeling down, depressed, or hopeless |  |  |  |  |
| 3. Trouble falling or staying asleep, or sleeping too much |  |  |  |  |
| 4. Feeling tired or having little energy |  |  |  |  |
| 5. Poor appetite or overeating |  |  |  |  |
| 6. Feeling bad about yourself or that you are a failure or have let yourself or your family down |  |  |  |  |
| 7. Trouble concentrating on things, such as reading the newspaper or watching television |  |  |  |  |
| 8. Moving or speaking so slowly that other people could have noticed. Or the opposite being so fidgety or restless that you have been moving around a lot more than usual |  |  |  |  |
| 9. Thoughts that you would be better off dead, or of hurting yourself |  |  |  |  |

**Part 3: Binge eating disorder**

| **1. During the last 3 months,** did you have any episodes of excessive overeating (i.e., eating significantly more than what most people would eat in a similar period of time)? | YES | NO |
| --- | --- | --- |
| **2.** Do you feel distressed about your episodes of excessive overeating? | YES | NO |

**NOTE: IF YOU ANSWERED “NO” TO QUESTION 1, YOU MAY STOP.**

**THE REMAINING QUESTIONS DO NOT APPLY TO YOU.**

| **Within the past 3 months…** | Never or Rarely | Sometimes | Often | Always |
| --- | --- | --- | --- | --- |
| **3. During your episodes of excessive overeating,** how often did you feel like you had no control over your eating (e.g., not being able to stop eating, feel compelled to eat, or going back and forth for more food)? |  |  |  |  |
| **4. During your episodes of excessive overeating,** how often did you continue eating even though you were not hungry? |  |  |  |  |
| **5. During your episodes of excessive overeating,** how often were you embarrassed by how much you ate? |  |  |  |  |
| **6. During your episodes of excessive overeating,** how often did you feel disgusted with yourself or guilty afterward? |  |  |  |  |
| **7. During the last 3 months,** how often did you make yourself vomit as a means to control your weight or shape? |  |  |  |  |

Thank you for your kind cooperation. (**Signature of the Interviewer)**
